# Two new species of the genus *Latouchia* Pocock, 1901 from southeast China (Araneae: Mygalomorphae: Halonoproctidae)

**DOI:** 10.3897/BDJ.9.e72456

**Published:** 2021-12-07

**Authors:** Shu-Yuan Zhang, Cheng-Bin Wang

**Affiliations:** 1 Engineering Research Center for Forest and Grassland Disaster Prevention and Reduction, Mianyang Normal University, 166 Mianxing West Road, Mianyang, China Engineering Research Center for Forest and Grassland Disaster Prevention and Reduction, Mianyang Normal University, 166 Mianxing West Road Mianyang China

**Keywords:** mygalomorph spider, Ummidiinae, *Latouchia*, taxonomy, Oriental Region

## Abstract

**Background:**

The genus *Latouchia* Pocock, 1901 (Araneae: Mygalomorphae: Halonoproctidae: Ummidiinae) includes 21 species and 1 subspecies occurring in southeast Eurasia. Just like other trapdoor spiders, the specimens of *Latouchia* are rare in collections, unless from targeted collecting.

**New information:**

Two new species of mygalomorph spiders, *Latouchiarufa* sp. n. from Guangdong, China and *L.yejiei* sp. n. from Hainan, China, are described and illustrated from both sexes. Diagnostic characters of the two species are provided.

## Introduction

Spiders of the genus *Latouchia* Pocock, 1901 (Araneae: Mygalomorphae: Halonoproctidae: Ummidiinae) are typical trapdoor-burrow dwellers. Recently, *Latouchia* was transferred from Ctenizidae to Halonoproctidae and it was considered as the sister group of *Conothele* + *Ummidia* clade (Godwin et al. 2018). The relative scarcity of specimens in museum collections might be the reason why this genus was rarely known in taxonomy (Haupt and Shimojana 2001). Decae (2019) and Decae et al. (2021) questioned the unity of *Latouchia* and Decae and Caranhac (2020) re-established *L.fossoria* Pocock, 1901 as the type species of the genus. For the fauna of China, eight species and one subspecies of *Latouchia* were recorded (World Spider Catalog 2021): *L.cornuta* Song, Qiu & Zheng, 1983; *L.davidi*（Simon, 1886); *L.formosensis* Kayashima, 1943; *L.formosensissmithi* Tso, Haupt & Zhu, 2003; *L.fossoria* Pocock, 1901; *L.hunanensis* Xu, Yin & Bao, 2002; *L.pavlovi* Schenkel, 1953; *L.typica* (Kishida, 1913) and *L.vinhiensis* Schenkel, 1963. In this study, two new species of *Latouchia* from southeast China are described and illustrated: *L.rufa* sp. n. from Guangdong and *L.yejiei* sp. n. from Hainan.

## Materials and methods

Specimens examined for this study were collected by excavation or by using pitfall traps. All specimens were preserved in 75%–100% ethanol and female genitalia were cleared with trypsase. Measurements were taken using a Leica M205A stereomicroscope in millimetres. The measurements of legs are shown as: total length (femur, patella, tibia, metatarsus and tarsus). Photographs were taken using the Leica M205A stereomicroscope, equipped with a DFC 550 CCD camera. The holotypes and paratypes are deposited in the insect collection of Mianyang Normal University, Mianyang, China (MYNU).

Abbreviations used in this paper: **ALE**, anterior lateral eyes; **AME**, anterior median eyes; **PLE**, posterior lateral eyes; **PME**, posterior median eyes.

## Taxon treatments

### Latouchia rufa

Zhang & Wang, 2021 sp. n.

98DBFE09-81AB-5F42-9ECF-4AD7A7DB1D3C

2F8E3CB3-3FBA-486F-A94B-36B7EB0034DA

#### Materials

- **Type status:** Holotype. **Occurrence:** recordedBy: Shu-Yuan Zhang; Ju Zhang; Bo Su; individualCount: 1; sex: male; **Location:** country: CHINA; stateProvince: Guangdong; verbatimLocality: Shenzhen City, Luohu District, Wutongshan National Park; verbatimLatitude: 22.5838°N; verbatimLongitude: 114.2216°E; **Event:** year: 2018; month: 4; day: 6; **Record Level:** institutionCode: MYNU
- **Type status:** Paratype. **Occurrence:** recordedBy: Shu-Yuan Zhang; Ju Zhang; Bo Su; individualCount: 5; sex: 1 male, 4 females; **Location:** country: CHINA; stateProvince: Guangdong; verbatimLocality: Shenzhen City, Luohu District, Wutongshan National Park; verbatimLatitude: 22.5838°N; verbatimLongitude: 114.2216°E; **Event:** year: 2018; month: 4; day: 6; **Record Level:** institutionCode: MYNU

#### Description

**Figure 1.**
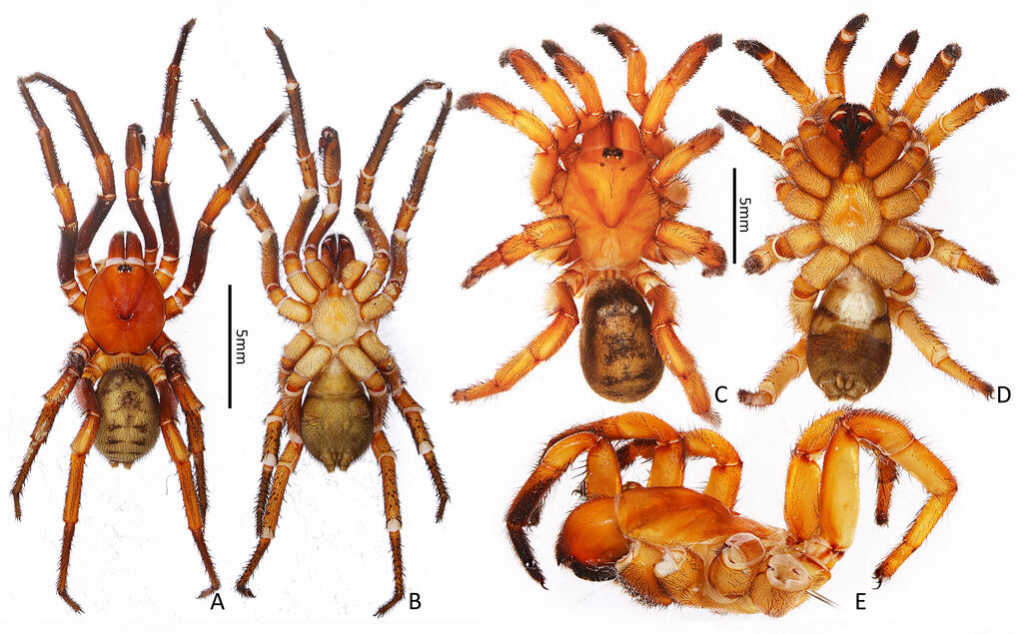
Habitus of *Latouchiarufa* sp. n. **A** male holotype, dorsal view; **B** male holotype, ventral view; **C** female paratype, dorsal view; **D** female paratype, ventral view; **E** female paratype, lateral view.

**Male (Fig. 1A and B).** Holotype (Ar.-MYNU-GDSZ0001). Total length 17.56 (not including chelicerae), chelicerae 3.17 long. Carapace 9.51 long and 8.59 wide, reddish-brown in ethanol (slightly darker when alive); rim of carapace black and with slight ridge, with a small number of setae; fovea U-shaped, with smooth flabalate thickening posteriorly; caput slightly raised, with numerous transverse grooves between ocular tubercle and fovea. Eyes in two rows, the front row slightly procurved; eye sizes and interdistances: AME 0.28, ALE 0.49, PME 0.26, PLE 0.38, AME–AME 0.14, ALE–ALE 0.64, AME–ALE 0.21, PME–PME 0.50, PME–PLE 0.12, ALE–PLE 0.09. Chelicerae reddish-brown, slightly darker than carapace, rastellum formed by 6–8 stout spines on a tubercle; promargin with 6–7 teeth, retromargin with 6 teeth. Labium 1.35 long and 1.70 wide, without cuspules. Maxillae 2.81 long and 1.78 wide, with 17 cuspules each. Sternum 5.31 long and 5.10 wide, reddish-brown, with large sigilla centrally. Several spinules present on a slightly sclerotised area between carapace and coxae III–IV. Opisthosoma 8.06 long and 5.91 wide, dorsally covered with five pairs of black marks and some irregular dark spots. Leg measurements: leg I 29.48 (9.21, 4.69, 7.36, 5.96, 2.26), leg II 25.02 (8.14, 4.03, 5.70, 5.12, 2.03), leg III 25.11 (6.39, 4.04, 4.32, 6.99, 3.37), leg IV 32.10 (8.27, 4.22, 7.52, 8.10, 3.99). Scopulae present on tarsi I–II and distal metatarsi I–II. Spines on prolateral tibia I–II hooked, the tip of hooked spine almost all upwards.

**Figure 2.**
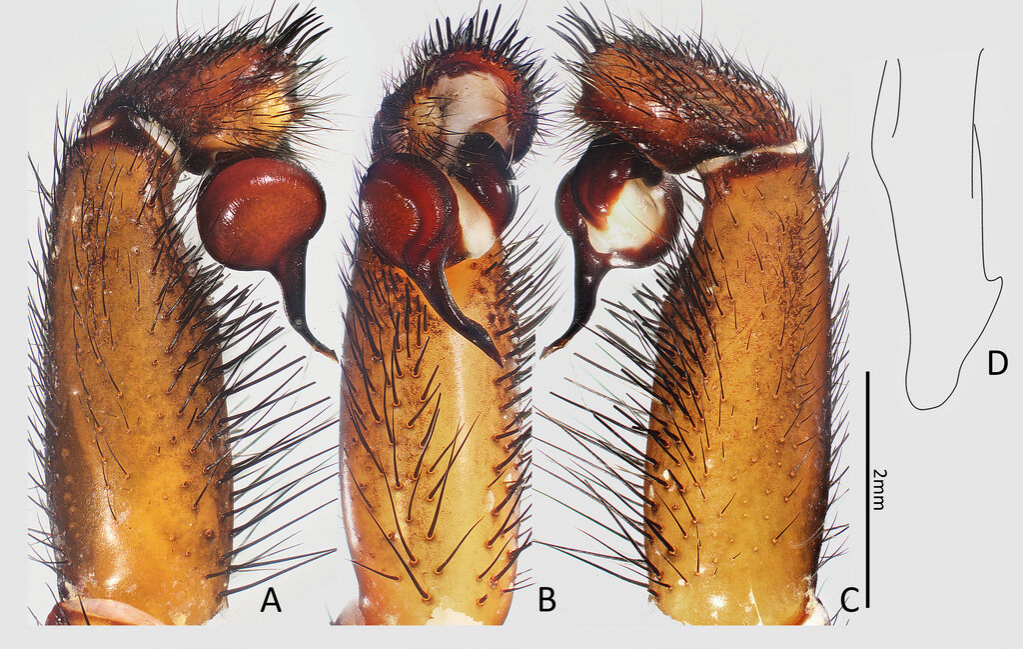
Left male palp of *Latouchiarufa* sp. n., holotype. **A** retrolateral view; **B** ventral view; **C** prolateral view; **D** tip of embolus.

Palp (Fig. 2A–D). Embolus medially curved, with a retrolateral keel; terminal part of embolus with a hooked apophysis dorsally. Palpal tarsus with ca. 10 xiphoidal spines distally; tibia with numerous bristles ventrally, without spine.

**Female (Fig. 1C–E).** One of paratypes (Ar.-MYNU-GDSZ0002). Total length 22.45 (not including chelicerae), chelicerae 4.78 long. Carapace 10.85 long and 9.00 wide, smooth, reddish-orange in ethanol (slightly darker when alive); fovea U-shaped; caput obviously raised. Eyes in two rows on tubercle; eye sizes and interdistances: AME 0.28, ALE 0.50, PME 0.22, PLE 0.49, AME-AME 0.21, ALE-ALE 0.73, AME-ALE 0.28, PME-PME 0.51, PME-PLE 0.12, ALE-PLE 0.11. Chelicerae robust, reddish-orange, promargin with 7 teeth, retromargin with 5–6 teeth; rastellum formed by 12 stout spines on a tubercle. Labium 1.47 long and 1.96 wide, without cuspules. Maxillae 3.70 long and 2.62 wide, with 16 cuspules each. Sternum 6.72 long and 6.08 wide, yellowish-orange, with large sigilla centrally. Several spinules present on a slightly sclerotised area between carapace and coxae III–IV. Opisthosoma 10.35 long and 7.29 wide, yellowish-brown, pattern as in male. Leg measurements: leg I 22.04 (7.38, 4.43, 4.54, 3.94, 1.75), leg II 18.29 (6.12, 3.59, 3.82, 3.00, 1.76), leg III 18.16 (5.44, 3.85, 2.68, 3.53, 2.66), leg IV 24.94 (7.74, 4.97, 4.88, 5.11, 2.24). Femora I–IV and patellae I–II without spines. Spines on palp, leg I and prolateral leg II almost all hooked.

**Figure 3.**
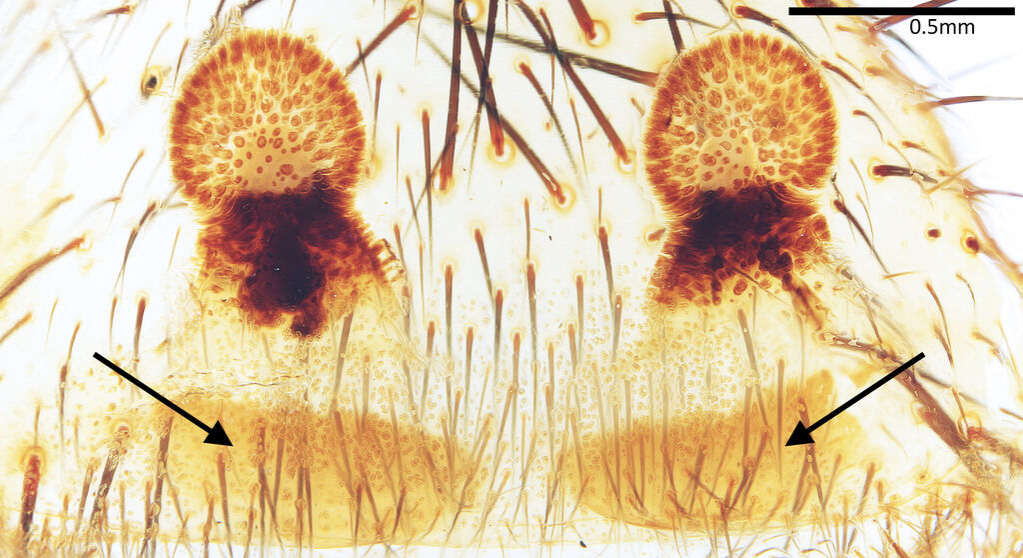
Vulva of *Latouchiarufa* sp. n., paratype, dorsal view. Arrows indicate basal plates.

Vulva (Fig. 3). Spermathecae suborbicular, with numerous glandular pores and an outer lateral protrusion; stalks short; basal plates present, slightly sclerotised.

#### Diagnosis

The male of this new species is similar to *Latouchiaformosensis* Kayashima, 1943 in general appearance, but it is distinguishable from the latter by the medially-curved embolus and the absence of a spine band on retrolateral side of palpal tibia (Fig. 2A–C) (vs. in *L.formosensis*, the embolus is relatively straight and median part is not curved; a band of short spines is present on retrolateral side of palpal tibia).

The new species also resembles *Latouchiaformosensissmithi* Tso, Haupt & Zhu, 2003 in the shape of terminal embolus (Fig. 2D) (both with a ridge proventrally) and in the arrangement of hooked spines on prolateral tibia II in males, but it can be distinguished from the latter by the following characters: in the lateral aspect of male palp, the width of the basal embolus is 2–3 times as wide as the tip (vs. in *L.formosensissmithi*, the basal embolus in the same aspect is 4–5 times as wide as the tip); in female, the spermathecae have an outer lateral protrusion.

#### Etymology

The specific name is Latin, means red, referring to the coloration of females. This adjective is in nominative case and feminine.

#### Distribution

China (Guangdong).

#### Field observations

**Figure 4.**
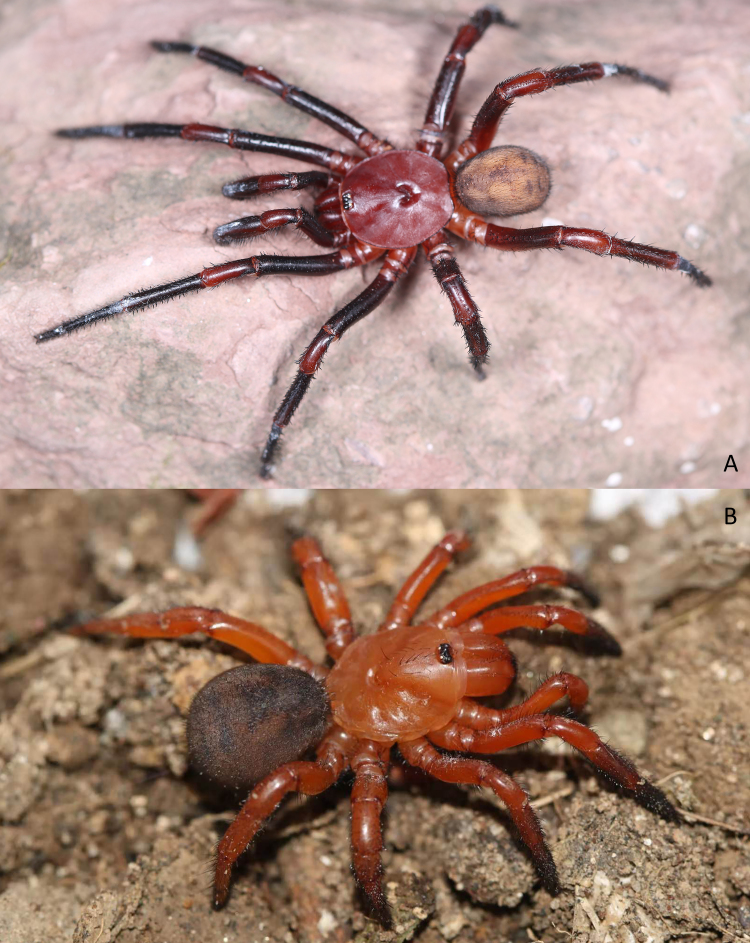
Living *Latouchiarufa* sp. n. **A** male (holotype); **B** female (paratype).

Living male and female of this new species as shown in Fig. 4.

### Latouchia yejiei

Zhang & Wang, 2021 sp. n.

AB24B9EB-352D-59EB-9EA6-AA7D8E7E604D

BCD8D8D7-9FF9-4802-B7F6-4C73C1D2BD13

#### Materials

- **Type status:** Holotype. **Occurrence:** recordedBy: Lin-Jie Gong; individualCount: 1; sex: male; **Location:** country: CHINA; stateProvince: Hainan; verbatimLocality: Ledong Li Autonomous County, Jianfengling National Nature Reserve, Road of Tianchi to Mingfenggu; verbatimElevation: 818 m; verbatimLatitude: 27.7406°N; verbatimLongitude: 117.6460°E; **Event:** year: 2018; month: 11; day: 23; **Record Level:** institutionCode: MYNU
- **Type status:** Paratype. **Occurrence:** recordedBy: Shu-Yuan Zhang; Zhe-Wen Ding; sex: 1 female; **Location:** country: CHINA; stateProvince: Hainan; verbatimLocality: Ledong Li Autonomous County, Jianfengling National Nature Reserve, Road of Tianchi to Mingfenggu; **Event:** year: 2020; month: 6; day: 30; **Record Level:** institutionCode: MYNU

#### Description

**Figure 5.**
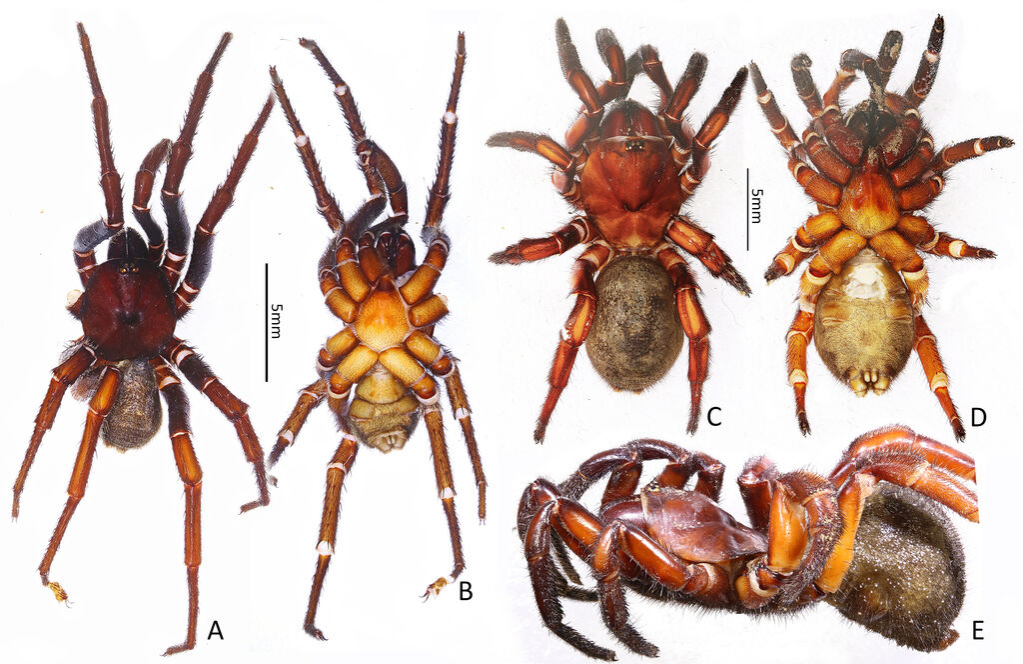
Habitus of *Latouchiayejiei* sp. n. **A** male holotype, dorsal view; **B** male holotype, ventral view; **C** female paratype, dorsal view; **D** female paratype, ventral view; **E** female paratype, lateral view.

**Male (Fig. 5A, B).** Holotype (Ar.-MYNU-HNLD0001). Total length 17.24 (not including chelicerae), chelicerae 4.58 long. Carapace 8.84 long and 8.25 wide, reddish-black in ethanol; rim of carapace black and with a slight ridge; fovea U-shaped, without any modification posteriorly; caput slightly raised, with numerous transverse grooves between ocular tubercle and fovea. Eyes in two rows, anterior row procurved; eye sizes and interdistances: AME 0.31, ALE 0.46, PME 0.32, PLE 0.47, AME-AME 0.20, ALE-ALE 0.76, AME-ALE 0.14, PME-PME 0.61, PME-PLE 0.07, ALE-PLE 0.27. Chelicerae reddish-black, rastellum formed by 10–12 stout spines on tubercle. Labium 1.29 long and 1.94 wide, without cuspules. Maxillae 3.32 long and 2.01 wide, without cuspules. Sternum 5.24 long and 4.71 wide, reddish-brown, with large sigilla centrally. Opisthosoma 8.41 long and 5.27 wide, dorsally yellowish-grey, without obvious pattern. Leg measurements: leg I 28.34 (8.17, 3.40, 7.28, 6.27, 3.22), leg II 26.19 (7.38, 3.26, 6.57, 5.94, 3.04), leg III 21.46 (4.64, 2.73, 4.24, 6.09, 3.76), leg IV 33.05 (8.47, 3.92, 7.99, 7.91, 4.76). Scopulae present on tarsi I–II and distal metatarsi I–II. Spines on anterior legs straight, some of them slightly hooked.

**Figure 6.**
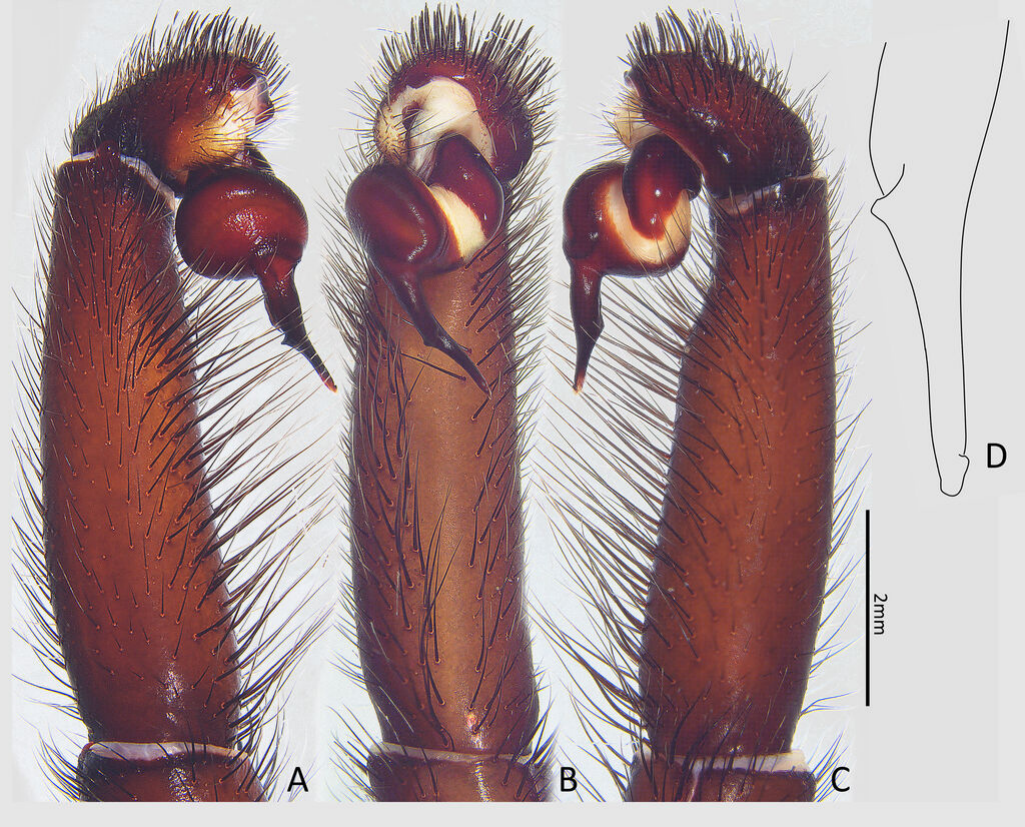
Left male palp of *Latouchiayejiei* sp. n., holotype. **A** retrolateral view; **B** ventral view; **C** prolateral view; **D** tip of embolus.

Palp (Fig. 6A–D). Tibia relatively slender, with numerous bristles ventrally; without spines. Embolus not obviously curved, with a median triangular apophysis proventrally.

**Female (Fig. 5C–E).** Paratype (Ar.-MYNU-HNLD0002). Total length 34.02 (not including chelicerae), chelicerae 5.03 long. Carapace 14.83 long and 12.90 wide, reddish-brown in ethanol (slightly darker when alive); fovea U-shaped; caput obviously raised. Eyes in two rows on tubercle, anterior row slightly procurved. Eye sizes and interdistances: AME 0.43, ALE 0.52, PME 0.46, PLE 0.48, AME–AME 0.27, ALE–ALE 0.41, AME–ALE 0.12, PME–PME 1.13, PME–PLE 0.09, ALE–PLE 0.41. Chelicerae robust; rastellum formed by ca. 15 stout spines on tubercle. Labium 2.73 long and 2.87 wide, with 7 cuspules. Sternum 8.22 long and 8.23 wide, reddish-brown, with large sigilla centrally. Opisthosoma 19.17 long and 13.31 wide, pattern as in male. Leg measurements: leg I 29.16 (10.20, 5.53, 6.89, 4.30, 2.24), leg II 24.40 (8.70, 4.19, 4.93, 4.58, 2.00), leg III 21.81 (7.64, 3.81, 3.79, 3.95, 2.62), leg IV 33.49 (10.30, 6.00, 6.52, 6.88, 3.79). Femora I–IV and patellae I–II without spines; most spines of anterior legs hooked.

**Figure 7.**
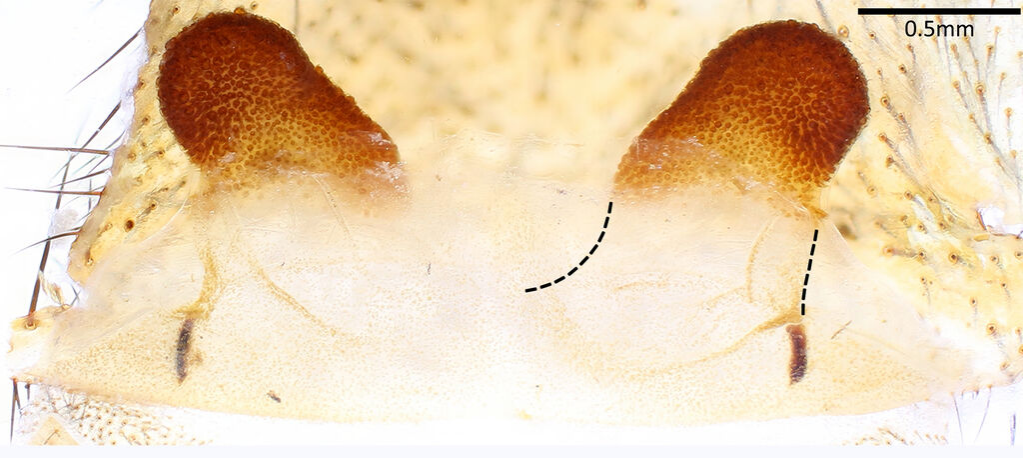
Vulva of *Latouchiayejiei* sp. n., paratype, dorsal view.

Vulva (Fig. 7). Spermathecae outward-tilted, not bulbous, with numerous glandular pores; stalks almost 1.5 times as wide as spermathecae.

#### Diagnosis

The female of *Latouchiayejiei* sp. n. resembles *L.bachmaensis* Ono, 2010, but it can be distinguished by the spermathecae being not bulbous; the stalks being broad, almost 1.5 times as wide as the spermathecae (vs. spermathecae slightly bulbous and stalks almost equal to spermathecae in *L.bachmaensis*). The male of the new species can be distinguished from other known male congeners by a triangular apophysis present on embolus proventrally (Fig. 6D) (It is not a triangular apophysis in *Latouchiaformosensissmithi* Tso, Haupt & Zhu, 2003, only a ridge proventrally).

#### Etymology

The specific name is given in honour of Mr. Ye-Jie Lin, who gave great help to this study. This noun is in genitive case and masculine singular.

#### Distribution

China (Hainan).

#### Field observations

**Figure 8.**
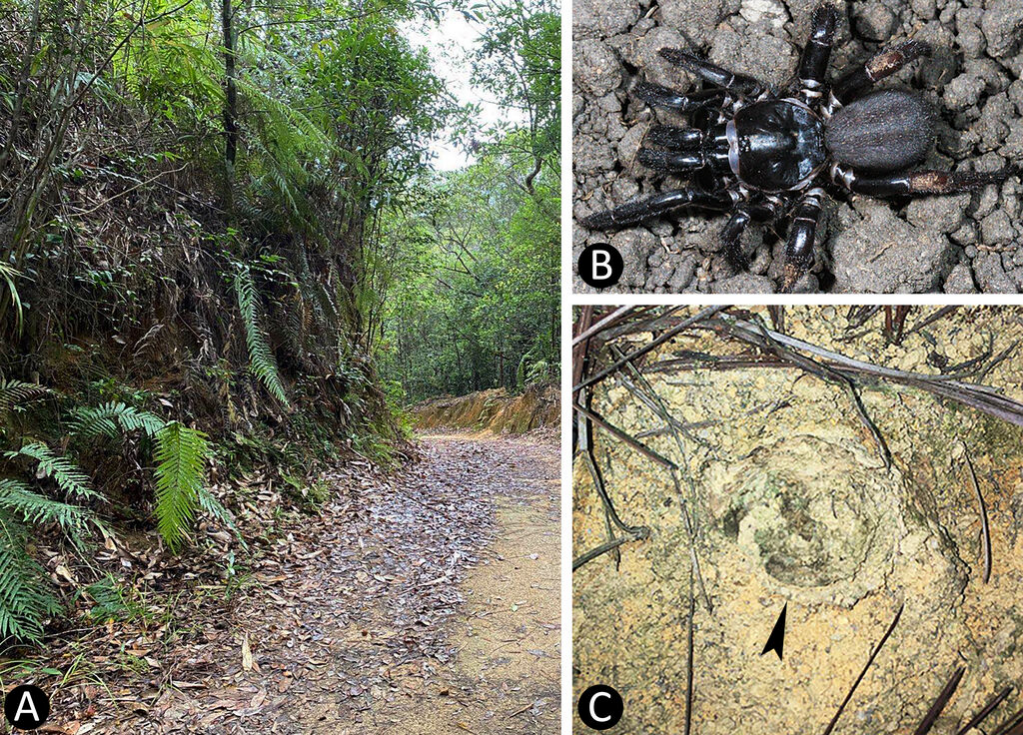
Field observations of *Latouchiayejiei* sp. n. at Jianfengling National Nature Reserve **A** habitat; **B** living female; **C** entrance of burrow, closed with trap door (indicated by arrow).

Habitat, living female and burrow as shown in Fig. 8.

## Supplementary Material

XML Treatment for Latouchia rufa

XML Treatment for Latouchia yejiei
